# Two maternal duplications involving the *CDKN1C* gene are associated with contrasting growth phenotypes

**DOI:** 10.1186/s13148-016-0236-z

**Published:** 2016-06-16

**Authors:** Susanne Eriksen Boonen, Andrea Freschi, Rikke Christensen, Federica Maria Valente, Dorte Launholt Lildballe, Lucia Perone, Orazio Palumbo, Massimo Carella, Niels Uldbjerg, Angela Sparago, Andrea Riccio, Flavia Cerrato

**Affiliations:** Department of Clinical Genetics, Aarhus University Hospital, 8200 Aarhus N, Denmark; Dipartimento di Scienze e Tecnologie Ambientali Biologiche e Farmaceutiche, Seconda Università degli Studi di Napoli, Caserta, Italy; Dipartimento di Biologia, Tigem, Napoli, Italy; Unità di Genetica Medica, IRCCS Casa Sollievo della Sofferenza, San Giovanni Rotondo, FG Italy; Department of Obstetrics and Gynecology, Aarhus University Hospital, 8200 Aarhus N, Denmark; Istituto di Genetica e Biofisica “Adriano Buzzati-Traverso”, Consiglio Nazionale delle Ricerche CNR, Napoli, Italy

**Keywords:** *CDKN1C*, Genomic imprinting, *KCNQ1OT1*, Microduplications, Growth disorders

## Abstract

**Background:**

The overgrowth-associated Beckwith-Wiedemann syndrome (BWS) and the undergrowth-associated Silver-Russell syndrome (SRS) are characterized by heterogeneous molecular defects affecting a large imprinted gene cluster at chromosome 11p15.5-p15.4. While maternal and paternal duplications of the entire cluster consistently result in SRS and BWS, respectively, the phenotypes associated with smaller duplications are difficult to predict due to the complexity of imprinting regulation. Here, we describe two cases with novel inherited partial duplications of the centromeric domain on chromosome 11p15 associated with contrasting growth phenotypes.

**Findings:**

In a male patient affected by intrauterine growth restriction and postnatal short stature, we identified an *in cis* maternally inherited duplication of 0.88 Mb including the *CDKN1C* gene that was significantly up-regulated. The duplication did not include the long non-coding RNA *KCNQ1OT1* nor the imprinting control region of the centromeric domain (KCNQ1OT1:TSS-DMR or ICR2) in which methylation was normal. In the mother, also referring a growth restriction phenotype in her infancy, the duplication was de novo and present on her paternal chromosome. A different *in cis* maternal duplication, 1.13 Mb long and including the abovementioned duplication, was observed in a child affected by Tetralogy of Fallot but with normal growth. In this case, the rearrangement also included most of the *KCNQ1OT1* gene and resulted in ICR2 loss of methylation (LOM). In this second family, the mother carried the duplication on her paternal chromosome and showed a normal growth phenotype as well.

**Conclusions:**

We report two novel *in cis* microduplications encompassing part of the centromeric domain of the 11p15.5-p15.4 imprinted gene cluster and both including the growth inhibitor *CDKN1C* gene. Likely, as a consequence of the differential involvement of the regulatory *KCNQ1OT1* RNA and ICR2, the smaller duplication is associated with growth restriction on both maternal and paternal transmissions, while the larger duplication, although it includes the smaller one, does not result in any growth anomaly.

Our study provides further insights into the phenotypes associated with imprinted gene alterations and highlights the importance of carefully evaluating the affected genes and regulatory elements for accurate genetic counselling of the 11p15 chromosomal rearrangements.

**Electronic supplementary material:**

The online version of this article (doi:10.1186/s13148-016-0236-z) contains supplementary material, which is available to authorized users.

## Findings

### Introduction

Less than 1 % of human genes are imprinted, that is, their expression is monoallelic and parent of origin-dependent as a result of epigenetic modifications acquired during gametogenesis [1]. Alterations of imprinted gene expression result in imprinting disorders (IDs) that are characterized by growth, metabolic, and developmental anomalies. Imprinted genes are generally organized in clusters that share regulatory *cis-*acting elements, such as enhancers and imprinting control regions (ICRs). The ICRs are 2–4-kb long genomic sequences characterized by repressive and permissive epigenetic marks on the opposite parental alleles. A large cluster of imprinted genes that is located on chromosome 11p15.5-p15.4 harbors two independent ICRs, H19/IGF2:IG (Intergenic)-DMR (also known as ICR1), and KCNQ1OT1:TSS (transcription start site)-DMR (also known as ICR2). ICR2 controls the imprinting of the centromeric domain. This region corresponds to the promoter of *KCNQ1OT1*, a long non-coding RNA that is transcribed antisense to *KCNQ1* and represses *in cis* the flanking imprinted genes on the paternal chromosome. These include *KCNQ1*, a member of the potassium channel KQT-family, and two genes with growth inhibitory properties, *CDKN1C* and *PHLDA2* [1–3].

Opposite genetic and epigenetic anomalies of the 11p15.5-p15.4 region result in the overgrowth-associated Beckwith-Wiedemann syndrome (BWS, MIM #130650) [4] and the undergrowth-associated Silver-Russell syndrome (SRS, MIM #180860) [5]. The BWS patients usually show one of the following defects: (1) gain of methylation (GOM) of ICR1 (5–10 % of the cases); (2) loss of methylation (LOM) of ICR2 (50 % of the cases); and (3) aberrant methylation of both ICRs due to segmental paternal uniparental disomy (UPD, 20 % of the cases) of chromosome 11. Conversely, the SRS patients frequently show ICR1 LOM (50 % of the cases); maternal UPD of chromosome 11p15 has been reported in only one case [1, 6]. *CDKN1C* variations affecting CDKN1C function can also cause these diseases. Maternally inherited loss-of-function mutations have been described in 5 % of the BWS patients (and 50 % of the familial cases) while gain-of-function mutations have been reported in the intrauterine growth restriction (IUGR)-associated IMAGe syndrome and in a single familial case of SRS [7, 8].

Deletions/duplications of chromosome 11p15.5-p15.4 have generally been reported in only 2–6 % of BWS and SRS patients [9], but a more recent study demonstrates an 8.4 % frequency in BWS patients [10]. Duplications encompassing the entire imprinted gene cluster are usually associated with BWS if paternally inherited and with SRS if maternally inherited. In addition, paternal duplication of the telomeric domain usually results in BWS [11, 12] and maternal duplication of the centromeric domain results in SRS [13, 14]. The contrasting phenotypes observed on maternal and paternal transmission of these chromosome alterations are likely caused by opposite deregulation of *IGF2* in the telomeric domain and *CDKN1C* and *PHLDA2* in the centromeric domain [15]. In the case of smaller duplications encompassing only a part of a single domain, the clinical outcome is difficult to predict because of the complex regulation of the 11p15 imprinted gene cluster.

Here, we describe two novel submicroscopic *in cis* duplications including part of the centromeric domain of the 11p15 imprinted gene cluster. The duplications extend 0.88 and 1.13 Mb from the middle of the *KCNQ1* gene toward the centromere, respectively. Despite both chromosome aberrations involve the *CDKN1C* gene and two out of the three putative enhancers [16], we find that only the smaller one is associated with growth restriction. The finding that the larger duplication also includes a hypomethylated ICR2 and part of *KCNQ1OT1* provides a possible explanation for the associated contrasting growth phenotypes.

## Results

**Fig. 1:**
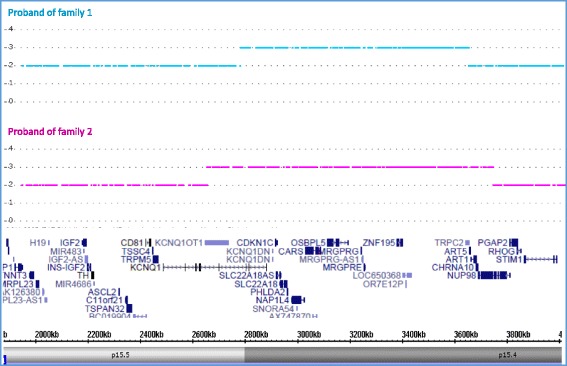
Extension of the 11p15.5-p15.4 duplications. Copy number analysis at chromosome 11p15 in the probands of the two families as determined by SNP array. The 0.88 Mb duplication of family 1 extends from chr11:2,742,159 to chr11:3,632,246 bp. The 1.13 Mb duplication of family 2 extends from chr11:2,656,311 to chr11:3,782,347 bp (GRCh37/hg19)

Two unrelated children with rare submicroscopic imbalances in the centromeric domain of the 11p15 imprinted gene cluster were identified and subjected to further laboratory analyses and clinical examination. In the proband of the first family (family 1), a duplication of about 0.88 Mb of chromosome 11p15.5-p15.4 was identified by comparative genomic hybridization (CGH) and single nucleotide polymorphism (SNP) array analyses. These methods allowed locating the telomeric breakpoint between chr11:2,739,336 and chr11:2,742,159 bp (GRCh37/hg19) within the intron 10 of *KCNQ1*, about 20-kb centromeric to ICR2, and the centromeric breakpoint between chr11:3,632,246 and chr11:3,632,370 bp in an intergenic region among the pseudogenes *LOC650368* and *TRPC2* (Fig. 1 and Additional file 1: Figure S1). In the proband of the second family (family 2), a duplication of 1.13 Mb was identified and its breakpoints defined by SNP array (Fig. 1). The telomeric breakpoint was identified inside intron 9 of *KCNQ1* gene, about 65 kb telomeric from the transcription start site of *KCNQ1OT1*, while the centromeric breakpoint was identified in intron 10 of the *NUP98* gene (arr[GRC37/hg19] dup(11)(p15.5-p15.4) (2,656,310x 2; 2,656,311-3,782,347x3; 3,782,492x2)).

### Family 1

The proband of family 1 was the third son of three children from unrelated parents. Pes equinovarus was observed by ultrasound scan by gestational age 16 + 2 weeks and an amniocentesis was obtained. The maternally inherited 11p15 duplication was identified. Due to this finding several ultrasound scans were performed during the pregnancy and IUGR was observed: −18 % at 30 + 6 weeks of gestation, −25 % at 32 + 5 weeks, −27 % at 34 + 5 weeks, and −30 % at 36 + 5 weeks. Due to IUGR induction of delivery was performed. He was born small for gestational age (SGA) at gestational age (GA) 37 + 6 weeks. His birth weight was 2070 g, (−3 SDS), birth length 44 cm (−3 SDS), and occipital frontal circumference (OFC) 30 cm (−2.5 SDS). The placenta weight (300 g, <third centile) was also reduced. Further, blood glucose was 1.4 mmol/l by delivery. He received treatment with intravenous glucose for 1 day. Afterwards he was only breast feed. The pes equinovarus was treated with plaster and tenotomy of the Achilles tendons at 45 days of age.

Physical examination at 14 months confirmed the growth restriction, in particular the short stature: weight 9.0 kg (−1.5 SDS), length 73 cm (−2.5 SDS), and OFC 45 cm (−1.5 SDS). The father, aged 40 years, was referred to be of normal stature (176 cm). The mother’s height was 163 cm and her weight is 59 kg at 38 years of age. It was referred that in childhood, she was very small and the general practitioner suspected she was a kind of a dwarf at 5–6 years of age.

The first son was born by GA 42 weeks with a birth weight of 3320 g (−0.5 SDS) and a birth length of 52 cm (average). By 14 years of age, his height was 168 cm (+1 SDS); the second son was born at 33 weeks of gestation. The spontaneous preterm delivery was caused by membrane rupture. His birth weight was 1575 g (−1.1 SDS), the birth length 41 cm (−1 SDS). Information on fetal growth parameters during pregnancy was not available. He was treated with intravenous glucose infusions age 1–3 days due to low blood glucose (blood glucoses at day 1: 1.9–2.6 mmol/l). By 7 years, his height was 127 cm (+1 SDS). He was affected by mild attention deficit hyperactivity disorder (ADHD).

**Fig. 2:**
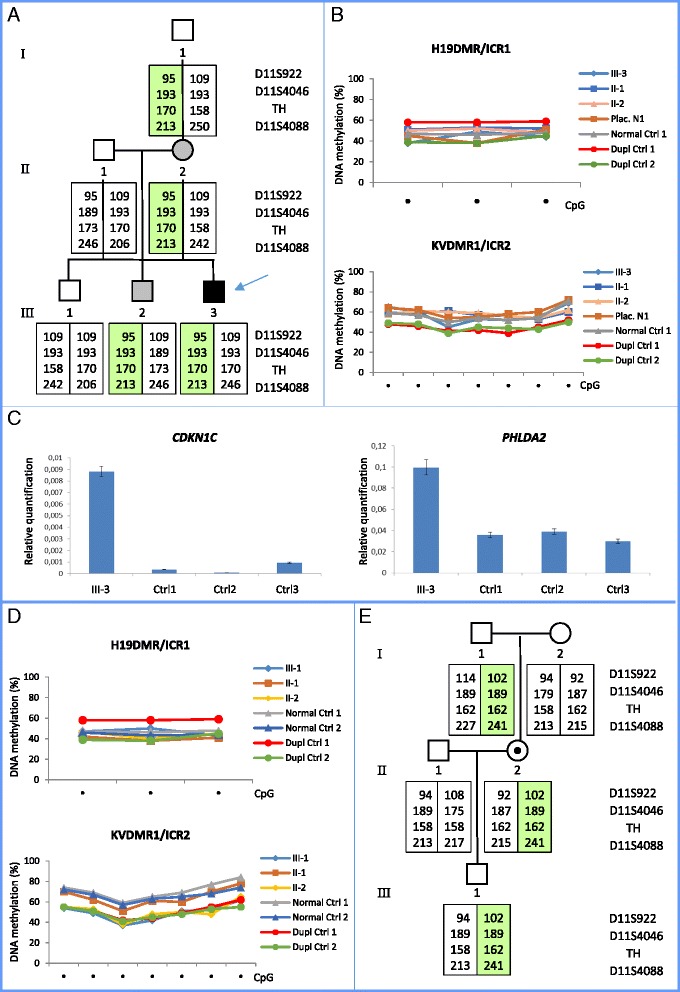
Characterization of the duplications. **a**–**c** Characterization of the 0.88 Mb duplication in family 1. **a** Analysis of 11p15 microsatellite markers showing the segregation of the duplication in three generations. The haplotype of the chromosome carrying the duplication is shadowed. *Gray color* of II-2 and III-2 indicates a growth restriction observed only in childhood. **b** DNA methylation analysis of ICR1 and ICR2 as determined by Pyrosequencing. Line chart reporting the methylation level (%) of seven CpGs of ICR2 and three CpGs of ICR1.The placenta DNAs of the proband (III-3) and a healthy control (Plac. N 1) and leukocyte DNAs of the parents (II-1 and II-2) and a healthy control (normal Ctrl 1) showed similar methylation patterns at both ICRs. Two BWS patients carrying a duplication of the entire domain [17, 18], Dupl Ctrl, have been analyzed as controls. **c** Real-time messenger RNA (mRNA) expression analysis of *CDKN1C* and *PHLDA2* normalized to the *GAPDH* control gene in the placenta cells of the proband (III-3) and three normal controls (Ctrl 1, 2, 3). Experiments were performed in triplicate and statistical significance determined by Student’s *t* test. **d**–**e** Characterization of the 1.13 Mb duplication in family 2. **d** DNA methylation analysis at ICR1 and ICR2 determined by Pyrosequencing in the trio as described in **b**. The proband (III-1) and his mother (II-2) show ICR2 methylation level similar to two BWS patients carrying ICR2 duplications (Dupl Ctrl 1 and 2) and lower than the father (II-1) and two healthy controls. ICR1 methylation of the proband and his parents is comparable to that of two healthy controls (normal Ctrl 1 and 2). **e** Analysis of 11p15 microsatellite markers showing a de novo paternal duplication in the mother and a maternally inherited duplication in the proband. The haplotype of the chromosome carrying the duplication is shadowed

The presence of the duplication was searched in the proband relatives by SNP array performed on DNA derived from buccal swab of the brothers and from blood of the parents and the maternal grandfather. The chromosome 11 duplication was identified in the mother and in her second son but not in the first son neither in the maternal grandfather (Additional file 2: Figure S2). By studying the segregation of the 11p15 haplotype by microsatellite analysis we demonstrated that in the mother the duplication was present on her paternal chromosome (Fig. 2a and Additional file 3: Figure S3). This suggests that the duplication originated very early in development, either in the gametes of the maternal grandfather or in the somatic cells of the mother.

To confirm the duplication and determine if it was present in *cis* or in *trans*, cells of the umbilical cord of the proband were analyzed by fluorescence in situ hybridization (FISH). A bacterial artificial chromosome (BAC) probe (RP11-11A9, chr11: 3236552-3356012, green signal in Additional file 4: Figure S4) hybridizing within the duplicated region and a BAC probe (RP11-876C12, chr11q22.3, red) located outside the duplication were used for the metaphase FISH (Additional file 4: Figure S4, top panel). FISH on interphase nuclei was performed by using the BAC clones RP11-11A9 (green) and RP11-81 K4 (red), both located within the duplicated region (Additional file 4: Figure S4, bottom panel). The absence of signals in chromosomes other than chromosome 11 in metaphase FISH, and the green-red-red-green sequence of the fluorescence signals, demonstrated the presence of an *in cis* duplication with inverted orientation.

To investigate the effect of the duplication on genomic imprinting, we analyzed the DNA methylation of ICR1 and ICR2 in the placenta cells of the proband by Pyrosequencing (Fig. 2b) and combined bisulfite restriction assay (COBRA; Additional file 5: Figure S5). With both methods, the proband showed a methylation profile comparable to that of three healthy controls in both ICR1 and ICR2. Normal ICRs methylation was also observed in the blood leukocytes of the parents. To look for a possible deregulation of the 11p15 imprinted genes, we analyzed the RNA levels of *CDKN1C* and *PHLDA2* in placenta cells. We found that *CDKN1C* expression was increased 10-fold (*P < 0.01*; Fig. 2c) and *PHLDA2* threefold in the proband when compared with three healthy controls (*P < 0.01*; Fig. 2c).

### Family 2

The male proband was the only child of non-consanguineous healthy parents. He was born by GA 37 + 5 weeks. Birth weight was 3350 g (−0.5 SDS), birth length 51 cm (average), and OFC 35 cm (+0.5 SDS). Apgar scores were 8/1, 8/5, and 8/10. Neonatal plasma glucose was normal. In the medical record, it is described that there was slight cranial asymmetry with left side of parietal and frontal region a little flat. The head was described as slight narrow, the nasal bridge as slightly wide, and there was strabismus and retention testis. Further, there were described bilateral dysplastic nails on third, fourth, and fifth toes. He was affected by Steno-Fallot Tetralogy, diagnosed on day 1 by echocardiography required because of a systolic murmur. Operation was performed by age 8 month. Neonatal ultrasound scans of cerebrum and kidneys were both normal. At 1 year old, he showed slight frontal bossing, slight hypoplasia of maxilla, slightly flaccid occiput, and bilateral single palmar creases. Neither umbilical hernia nor ear lobe creases were observed. At 8 and 20 months of age, the auxological parameters were still close to the average: 8 month: weight 9.2 kg (+0.5 SDS), length 71.5 cm (+0.5 SDS) and OFC 44 cm (−0.5 SDS); 20 month: weight 11.3 kg (−0.5 SDS), length 84.5 cm (−0.5 SDS) and OFC 46.3 cm (−1.5 SDS). The psychomotor development was normal.

The mother was 35 years old, with normal phenotype except for the presence of bilateral ear lobe creases. Her height was 170 cm and weight was 56.5 kg. She was born by GA 41 + 5 weeks, with the birth weight 3740 g (+0.5 SDS), birth length 53 cm (+1 SDS), and OFC 35.5 cm (+1.5 SDS).

Copy number and DNA methylation of the chromosome 11p15.5 region were first analyzed by MS-MLPA. Increased hybridization signal at ICR2 and *KCNQ1* exons 13–17 and slight loss of ICR2 methylation were identified in the proband and his mother, while ICR1, *IGF2*, *H19*, and control probes showed normal copy number and methylation status (Additional file 6: Figure S6), indicating the presence of an inherited partial duplication of the 11p15.5-p15.4 imprinted gene cluster.

To better define the DNA methylation abnormality of the 11p15.5-p15.4 region in the proband and his mother, the methylation levels of the ICRs were determined by pyrosequencing in the trio. As shown in Fig. 2d, the methylation profiles of both ICRs were normal in the father, while the proband and his mother showed normal methylation of ICR1 but hypomethylation of ICR2 at a level comparable with other previously described ICR2 duplication carriers [17, 18]. The allele-specific methylation analysis could not be performed because of the absence of polymorphisms in the ICR2 sequence. Nevertheless, the observed hypomethylation suggests that the duplicated ICR2 fails to acquire or maintain the maternal imprints in the proband.

The inheritance of the duplicated region in the proband, his parents and maternal grandparents, was determined by analyzing the 11p15 microsatellite markers. The segregation and signal intensity of the D11S4088 marker, located in the duplicated region, confirmed that the duplication was maternally inherited in the proband and demonstrated that it originated de novo from the paternal chromosome in the mother (Fig. 2e and Additional file 7: Figure S7). The markers, D11S4046, D11S922, and TH, did not show any allelic imbalance in the proband and his mother, consistent with their localization outside of the duplicated region (Additional file 7: Figure S7).

To determine the chromosomal location of the duplicated region, the cultured blood leukocytes of the proband were analyzed by FISH. The BAC probes hybridizing within the duplicated region, RP11-11A9 (chr11: 3236552-3356012, green) and RP11-699D10 (chr11: 2.9–3.04 Mb, red), were used. As in family 1, the results of the metaphase FISH indicated that the duplication was *in cis* (Additional file 8: Figure S8, top panel), while the FISH on interphase nuclei demonstrated the inverted orientation of the duplication (Additional file 8: Figure S8, bottom panel).

## Discussion

Maternal duplications of the centromeric domain of the 11p15 imprinted gene cluster generally result in SRS phenotype. In this study, we describe two familial cases with overlapping maternal duplications that partially affect the centromeric domain and show contrasting growth phenotypes.

**Fig. 3:**
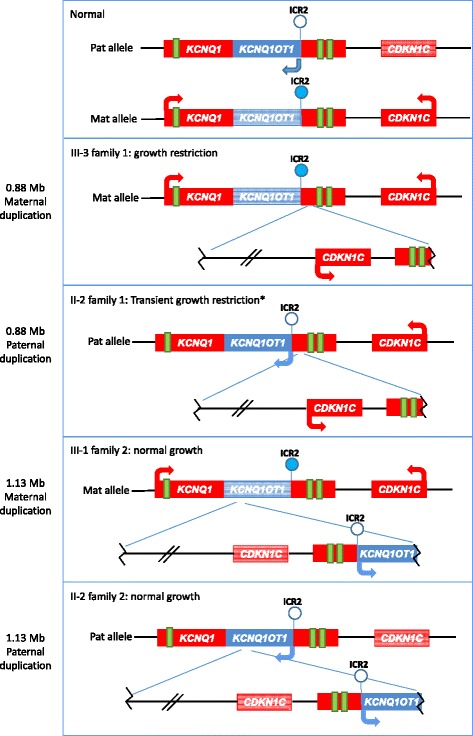
Observations and predictions concerning the two duplications. Diagram summarizing the molecular and clinical phenotypes of the subjects under study. The duplicated regions are depicted separately from the chromosomes and connected to the breakpoints by *blue lines*. The inverted orientation of the duplications is shown. Representative imprinted genes are shown in *blue* if paternally expressed or in *red* if maternally expressed. *Light colors* are used to indicate repressed genes, *full colors* for active genes. *Arrows* indicate the orientation of transcription. *Filled lollipops*: methylated ICR2; *open lollipops*: non-methylated ICR2. *Green rectangles*: putative *CDKN1C* enhancers as reported in [16]. Note that in III-1 of family 2 it is likely that only one copy of *CDKN1C* is silent, but it is not possible to determine which is silent and which is active. Asterisk represents growth restriction was observed only in childhood

Both the rearrangements described in this study duplicate the *CDKN1C* gene and two of its putative enhancers on the maternal chromosome 11p15 [16]. However, only the 0.88 Mb duplication (family 1) is associated with growth restriction. The most likely explanation of this discrepancy is the presence of *KCNQ1OT1* and an unmethylated ICR2 in the 1.13 Mb but not in the 0.88 Mbp duplication (Fig. 3). Expression of *KCNQ1OT1* resulting from ICR2 hypomethylation likely leads to down-regulation of the duplicated *CDKN1C* and *PHLDA2* in the family 2 proband. His normal growth phenotype suggests that the *KCNQ1OT1* transcript is unable to silence both copies of *CDKN1C* on the maternal chromosome (Fig. 3). Conversely, the normally methylated ICR2 in the family 1 proband results in *KCNQ1OT1* repression and over-expression of the duplicated *CDKN1C* and *PHLDA2*. Similarly, *CDKN1C* over-expression probably also occurs in the cases with larger maternal duplications maintaining ICR2 methylation [13–15].

Both the probands mothers are carriers of the duplications but on their paternal chromosomes. The family 1 mother (II-2) was growth restricted during her infancy. This phenotype likely results from *CDKN1C* expression on both maternal and paternal 11p15 chromosomes (Fig. 3). No *CDKN1C* deregulation is expected instead in the mother of family 2 because of the *KCNQ1OT1* duplication and ICR2 hypomethylation.

Maternal duplication of the entire centromeric domain or the entire 11p15 imprinted gene cluster is generally associated with clinical features of SRS and quite severe growth restriction (birth weight and length z scores −2.5/−7 SDS; postnatal growth restriction −2.5/−6.4 SDS; [19]). In contrast, the family 1 proband showed limited growth restriction and no other characteristics of SRS. Also, the occurrence of a compensatory growth later in development (he was 14 months old at the last examination) cannot be excluded. An even milder phenotype was observed in the second brother (III-2) of the proband, who is also a carrier of the duplication. He was of short stature in the first 3 years of life (height around 20–25th centile: 41 cm at birth, 65 cm (−1.5 SDS) at 6 months, 89 cm at 29 months (−0.5 SDS)), had hypoglycemia at birth, and was affected by ADHD. The attenuated phenotypes may be due to the limited extension of the 0.88 Mb duplication into the centromeric domain leaving out some of the putative *CDKN1C* enhancers (Fig. 3). It is worth to mention, however, that a mild SRS-like phenotype with ADHD was recently reported associated with a 1.9 Mb maternal duplication encompassing the entire 11p15.5 cluster [19].

Only a few other duplications encompassing partially the centromeric domain of the 11p15 imprinted gene cluster have been described so far. Two of these are 50 and 160 kb long, respectively, and both were associated with BWS upon maternal transmission. The 50-kb duplication spans from intron 1 to intron 2 of *KCNQ1* and was associated with ICR2 hypomethylation [9]. The 160 kb duplication spans from intron 9 to exon 15 of *KCNQ1* and included a non-methylated copy of ICR2 and the 5′ part of the *KCNQ1OT1* gene [17]. In both cases, the BWS phenotype likely results from the expression of the maternal *KCNQ1OT1* allele causing *CDKN1C* repression *in cis*. A complex 277 Kb rearrangement on the paternal chromosome 11p15 has been recently described associated with SRS [20]. In this case, a small portion of *KCNQ1OT1* and the entire *CDKN1C* gene were duplicated, but these duplications were discontinuous and did not include ICR2. In this case, the SRS phenotype likely results from the unregulated expression of *CDKN1C* on the paternal chromosome.

The 1.13-Mb duplication described in this study has some similarities with the 160 kb duplication associated with BWS [17]. Both rearrangements are present *in cis* and in inverted orientation and include a duplicated incomplete but likely functional copy of *KCNQ1OT1* and a hypomethylated ICR2. However, the former rearrangement is more extended toward the centromere and includes *CDKN1C*. The consequence is that the 160 kb duplication results in reduced *CDKN1C* expression and BWS, while the 1.13-Mb duplication is associated with normal growth and likely normal *CDKN1C* level (Fig. 3).

The Tetralogy of Fallot (TOF) affecting the proband of family 2 is a severe congenital heart malformation (MIM #187500) with both environmental and genetic etiology. The genetics of TOF is complex and involves many loci, but studies performed on large cohorts have not identified any strict association with defects on chromosome 11p [21, 22]. Nevertheless, a few cases of paternal 11p15 duplications with BWS and cardiac malformations including TOF have been described [23–25]. Therefore, the involvement of 11p15 genes in the etiology of rare TOF cases deserves further investigations.

Ear lobe creases are a common sign of BWS [1]. Although more frequently associated with *CDKN1C* alterations, they can be found in BWS cases with other types of 11p15.5 molecular defect including paternal duplications. The finding of such sign in the mother of family 2 proband is intriguing and may be due to altered expression of some 11p15.5 genes or may be coincidental and have different causes.

In summary, our study is an example of how the analysis of the small copy number variation (CNVs) affecting the imprinted gene clusters can increase our understanding on the imprinting regulatory mechanisms and help to predict the clinical phenotypes resulting from such type of rearrangements.

## Methods

### Biological samples

DNA from peripheral blood leukocytes of all the family members was extracted by an automated Chemagic Magnetic Separation Module (PerkinElmer, Waltham, MA, USA). DNA from buccal swab samples of the relatives of the proband from family 1 was extracted by using the Maxwell 16 LEV Buccal Swab DNA kit (Promega, Madison, WI, USA).

Cells from umbilical cord and placenta of the proband from family 1 and three normal controls were cultured in BIOAMF^TM^-3 (BI-USA Inc., Cromwell, CT, USA). DNA from cell culture of placenta and umbilical cord and amniotic fluid cells was extracted directly using the Maxwell 16 LEV Blood DNA kit (Promega, Madison, WI, USA). RNA was extracted by using TRIzol reagent (Life technologies, Darmstadt, Germany), according to the protocol of the manufacturer.

All the genetic analyses were performed after informed consent had been obtained. All the clinical and research have been done following the ethical rules of the Danish and Italian law.

### Growth parameters

Z scores of each member under study were calculated referring to standard growth rate of Denmark.

### Copy number variation detection

*MS-MLPA*. MS-MLPA was performed on genomic DNA of the proband from family 2, his parents and maternal grandparents. The SALSA MS-MLPA kit ME030-C3 for BWS–SRS (MRC-Holland, Amsterdam, the Netherlands) was used following the manufacturer’s instructions. The amplified products were separated by capillary electrophoresis using ABI 3130 Genetic Analyzer (Applied Biosystems, CA, USA). Data was analyzed using the built-in MS-MLPA tool of the software Genemarker v 2.2.0 (Softgenetics, USA).

#### CGH array

The samples were analyzed using the SurePrint G3 Human CGH microarray 180k (Agilent Technologies Inc., Santa Clara, CA, USA). Sample and reference genomic DNA (500 ng) were labelled with Cy5 (reference) or Cy3 (specimen) using the Sure Tag Complete DNA labelling Kit (Agilent Technologies Inc.) and purified as described in the manufacturer’s protocol. Labelled sample and reference DNA were pooled, and 5-μl human COT-1 DNA (1 mg/ml), 10× blocking agent, and 2× hybridization buffer were added. Hybridization was performed for 24 h at 65 °C. Scanning and image acquisition were carried out using an Agilent microarray scanner and microarray image files were analyzed using CytoGenomics, version 2.9 (Agilent Technologies Inc.). Copy number was determined using the adm-2 algorithm and profile deviations consisting of four or more neighboring oligonucleotides were considered genomic aberrations. The resolution is thus approximately 50 kb.

Detected copy number gains or losses were compared with our in-house database of CNVs and with public CNV databases (Database of Genomic Variants: http://dgv.tcag.ca/dgv/app/home; Decipher: http://decipher.sanger.ac.uk; ISCA: http://clinicalgenome.org/).

#### SNP array

Whole-genome copy number variation (CNV) analysis was carried out using the CytoScan HD array platform (Affymetrix, Santa Clara, CA). This array contains more than 2.6 million markers for copy number analysis and approximately 750,000 SNPs that fully genotype with greater than 99 percent accuracy. The CytoScan HD assay was performed starting with 250 ng DNA as previously described [26]. Both quality control step and copy number analysis were performed using the Chromosome Analysis Suite Software version 2.0. The raw data file (.CEL) was normalized using the default options; an unpaired analysis was performed using as baseline 270 HapMap samples in order to obtain copy numbers value from .CEL files; while the amplified and/or deleted regions were detected using a standard Hidden Markov Model (HMM) method. Karyotype was designated according to ISCN 2013, and base pair position was derived from the University of California Santa Cruz (UCSC) Genome Browser (http://genome.ucsc.edu/cgi-bin/hgGateway), build GRCh37 (hg19).

#### Microsatellite analysis

D11S4088 short tandem repeat (STR) marker mapping to the duplicated region and TH, D11S4046 and D11S900 STR mapping at the 11p15.5-4 region outside the duplication, were analyzed in the probands and their relatives to verify the origin of duplication and follow the segregation through the three generations. Primers specific for the STR were obtained from NCBI Genome Database together with the PCR conditions. PCR amplification of 100-ng DNA was done using forward primer end labelled with Fam or Hex. Twenty-eight cycles of PCR products were run on the fluorescent capillary system ABI 3130XL. Data were analyzed using GeneMapper Software.

Microsatellites of chromosome 7 (D7S657, D7S502, D7S686, D7S1830) were also analyzed to exclude the UPD7 associated with the 10 % of the SRS cases (data not shown).

### Fluorescence in situ hybridization

FISH analysis was performed on metaphase or interphase nuclei spread from PHA-stimulated umbilical cord cell culture (proband, family 1) and peripheral blood leukocytes (proband, family 2) using standard procedures. The RP11-699D10 (red) and RP11-11A9 (green) BlueFISH probes (Illumina) targeting the 11p15.4 duplicated region were used for FISH analyses of proband of family 1. RP11-179B7 (red) on 11q22.3 served as control for chromosome 11 to exclude a translocation defect. RP11-11A9 (green) and the BAC clone RP11-81 K4 DNA labelled with red fluorophore using a non-enzymatic nucleic acid labelling method (ULSTM, Kreatech Diagnostics Amsterdam, The Netherlands) were used as probes for the interphase FISH on proband of family 2. The probes used for the metaphase FISH were RP11-81K4 (11p15.5-15.4, green) and RP11-876C12 (11q22.3, red). The chromosomes and nuclei were counterstained with DAPI. Hybridizations were analyzed using a Leica DMRB microscope (Leica Microsystems A/S, Wetzlar, Germany) or a Nikon Eclipse-1000 epifluorescence microscope (Nikon Instruments, Tokyo, Japan). Images captured and elaborated using the ISIS software v. 5.1 (MetaSystems GmbH, Altlussheim, Germany) or the Genikon systemv. 3.8.5 (Nikon Instruments, Tokyo, Japan).

### DNA methylation analysis

Two micrograms of genomic DNA extracted from cells/tissues was treated with sodium bisulfite by using the EpiTect Bisulfite kit (Qiagen-Italia, Milan, Italy) following the manufacturer’s protocol. The converted DNA was analyzed by COBRA and Pyrosequencing.

#### COBRA

Bisulfite-treated DNA was amplified with primers specific for CTCF target site 1 of ICR1 and ICR2. The PCR products were then digested with *Bst*UI (CGCG) and the digestion products were run on a polyacrylamide gel to separate the digested (methylated) from the undigested (non-methylated) bands. The percentage of methylation was calculated by computer quantitation of the gel following exposure to phosphorimager. Primers sequences, PCR, and restriction enzyme reaction conditions were previously described [27, 28].

#### Pyrosequencing

In order to obtain more quantitative DNA methylation data, pyrosequencing was performed to assess methylation at seven CpGs within ICR2 and three CpGs within ICR1, as control. Primers and PCR conditions were previously described [29] (KvDMR1-F 5′-TTAGTTTTTTGYGTGATGTGTTTATTA-3′ and KvDMR1-R 5′-Biotin/CCCACAAACCTCCACACC-3′; for sequencing: KvDMR1-S 5′-TTGGTAGGTATAGAAATTGGGG-3′) and [30] (H19DMR-CTCF3 F 5′-TTGGTAGGTATAGAAATTGGGG-3′ and H19DMR-CTCF3R 5′-Biotin/ACACYTAACTTAAATAAC-3′; for sequencing: H19DMR-CTCF3 S2 5′-GTGGATTTAAAAGTGGT-3′). Sequencing of 10 μl of PCR product was carried out on a PSQ 96MD system with the PyroGold SQA Reagent Kit (Qiagen-Italia, Milan, Italy), and results were analyzed using the Q-CpG software (V.1.0.9Pyrosequencing).

### Gene expression analysis

About 1 μg of total RNA extracted from placenta and umbilical cord cultured cells was treated with RNase-free DNase, and first-strand complementary DNA (cDNA) was synthesized using Quantitech Reverse Transcription Kit (Qiagen-Italia, Milan, Italy), according to the protocol of the manufacturer. *CDKN1C* expression was examined by SYBR Green quantitative real-time PCR (Power SYBR Green Master Mix Applied Biosystems, Foster City, CA, USA). Reactions were run on ABI PRISM 7500 using the default cycling conditions. Relative expression was determined using the ΔΔ*C*_T_ method, and gene expression values were normalized to the expression of the *GAPDH* reference gene. The primers used are *CDKN1C* For 5′- AGAGATCAGCGCCTGAGAAG-3′ and *CDKN1C* Rev 5′-CACCTTGGGACCAGTGTACC-3′ [17]; *GAPDH* For 5′-CACCATCTTCCAGGAGCGAG-3′ and *GAPDH* Rev 5′-TCACGCCACAGTTTCCCGGA-3′.

## Abbreviations

BAC, bacterial artificial chromosome; BWS, Beckwith-Wiedemann syndrome; CGH, array comparative genomic hybridization; COBRA, combined bisulfite restriction assay; Ctrl, control; Dupl, duplication; FISH, fluorescence in situ hybridization; GOM, gain of methylation; ICR, imprinting control region; ID, imprinting disorders; IG-DMR, intergenic differentially methylated region; LOM, loss of methylation; MS-MLPA, methylation-specific multiplex-ligation-dependent amplification probe assay; OFC, occipital frontal circumference; SDS, standard deviation score; SNP, single nucleotide polymorphisms; SRS, Silver-Russell syndrome; TSS-DMR, transcription start site—differentially methylated region; UPD, uniparental disomy

## Additional files

Additional file 1: Figure S1. Comparative genomic hybridization analysis performed on DNA of the proband from family 1. The extension, the genomic localization (GRC h37/hg19) and the genes included in the duplication are shown. (PDF 54 kb) Additional file 2: Figure S2. Single nucleotide polymorphism analysis of genomic DNA from relatives of family 1. I-1 = maternal grandfather, II-2 = mother, III-1 = first brother of the proband, and III-2 = second brother of the proband. Note that the duplication is present in II-2 and III-2 but not in I-1 and III1. (PDF 127 kb) Additional file 3: Figure S3. Segregation of the haplotype associated with the duplication in family 1. Note that the haplotype associated with the duplication segregates from I-1 to II-2, III-2, and III-3 and that only the D11S4088 marker shows allelic imbalances in II-2, III-2, and III-3. (PDF 208 kb) Additional file 4: Figure S4. FISH analysis on metaphase nuclei (top panel) of cultured cells derived from the umbilical cord of the proband of family 1 by using BAC probes for 11p15.5-15.4 (RP11-81K4, 2798699-2970438, green) and 11q22.3 (RP11-876C12, 103,804,669-103,982,517, red). The green signal on both homologues is visible only at chr11p, demonstrating the presence of an *in cis* duplication and excluding an unbalanced translocation. FISH analysis on interphase nuclei (bottom panel) using the BACs RP11-11A9 (3,236,552-3,356,012, green) and RP11-81K4 (red), hybridizing within the duplication. Note that single and duplicated signals can be seen on the two homologues, respectively. The green-red-red-green order of the duplicated signals indicates that the duplication is inverted. (PDF 51 kb) Additional file 5: Figure S5. DNA methylation analysis of ICR1 and ICR2 in family 1, determined by combined bisulphite restriction assay (COBRA). The placenta DNAs of the proband and three healthy controls, the peripheral blood DNAs of the proband parents, one control, and two BWS patients (Dupl Ctrl) carrying a duplicated unmethylated ICR2 [15, 16]. Note that the proband (III-3) and his parents show normal methylation at both ICR1 and ICR2. Unme = non-methylated band, me = methylated band. (PDF 66 kb) Additional file 6: Figure S6. DNA methylation (top) and copy number (CN, bottom) analyses at 11p15 region in family 2, determined by MS-MLPA. The histograms represent the normalized DNA methylation of ICR1 and ICR2 and CN of the genomic region spanning from the *NSD1* to *KCNQ1* gene. The CN range that is considered normal is shadowed. Note that methylation of ICR2 is abnormally low while CN values of ICR2 and *KCNQ1* exon 13-17 are abnormally high in the proband and his mother. (PDF 130 kb) Additional file 7: Figure S7. Note that the haplotype associated with the duplication segregates from I-1 to II-2 and III-1 and that only the D11S4088 marker shows allelic imbalances in II-2 and III-1. (PDF 141 kb) Additional file 8: Figure S8. FISH analysis on metaphase nuclei (top panel) of cultured cells derived from peripheral blood leukocytes of the proband of family 2 by using BAC probes for 11p15.5-15.4 (RP11-11A9, 3,236,552-3,356,012, green) and 11q22.3 (RP11-179B7, 104,298,339-104,459,797, red). The green signal on both homologues is visible only at chr11p, demonstrating the presence of an *in cis* duplication and excluding an unbalanced translocation. FISH analysis on interphase nuclei (bottom panel) using the BACs RP11-699D10 (2.9–3.0 Mb, red) and RP11-11A9 (green), hybridizing within the duplication. Note that single and duplicated signals can be seen on the two homologues, respectively. The red-green-green-red order of the duplicated signals indicates that the duplication is inverted. (PDF 52 kb)
