# Supplementary figures and images for: Two maternal duplications involving the CDKN1C gene are associated with contrasting growth phenotypes
Source: Clin Epigenetics. 2016 Jun 16;8:69. doi: 10.1186/s13148-016-0236-z (PMC4910218; doi:10.1186/s13148-016-0236-z)

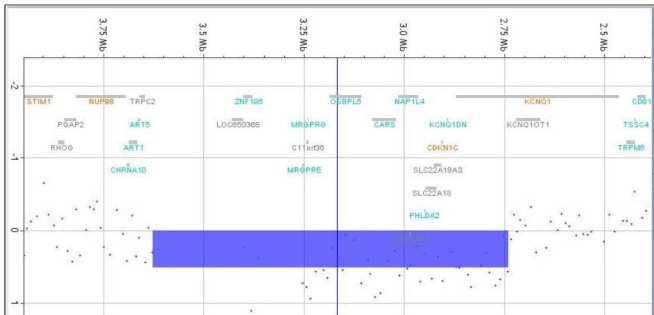

Additional file 1: Figure S1

Supplement: Additional file 1: Figure S1. — Comparative genomic hybridization analysis performed on DNA of the proband from family 1. The extension, the genomic localization (GRC h37/hg19) and the genes included in the duplication are shown. (PDF 54 kb) [file 13148_2016_236_MOESM1_ESM.pdf]

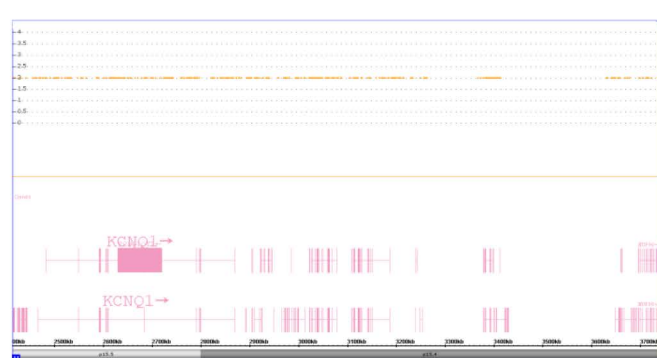

I1

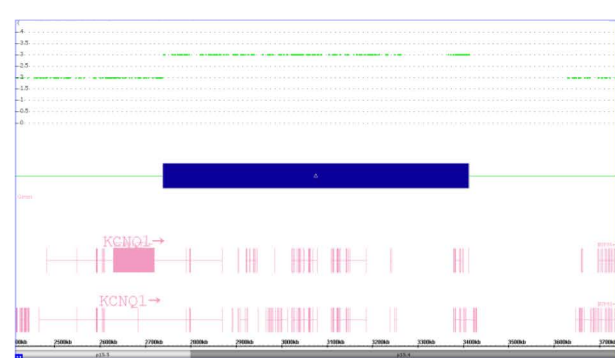

I12

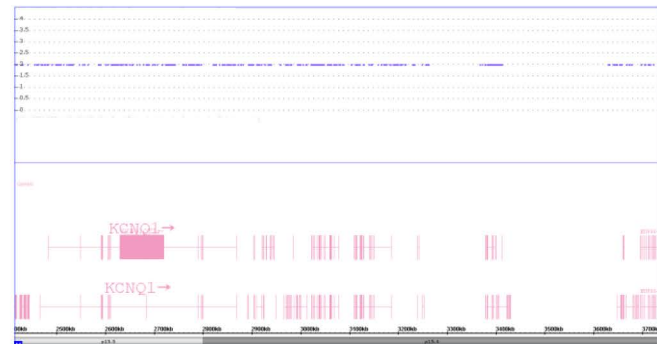

III1

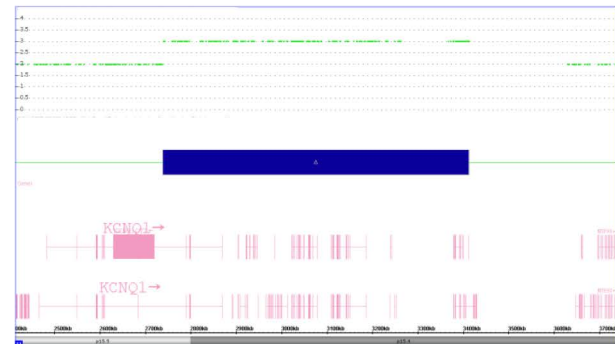

III2

Supplement: Additional file 2: Figure S2. — Single nucleotide polymorphism analysis of genomic DNA from relatives of family 1. I-1 = maternal grandfather, II-2 = mother, III-1 = first brother of the proband, and III-2 = second brother of the proband. Note that the duplication is present in II-2 and III-2 but not in I-1 and III1. (PDF 127 kb) [file 13148_2016_236_MOESM2_ESM.pdf]

## D11S922

I-1

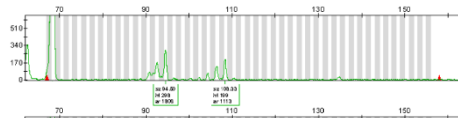

II-1

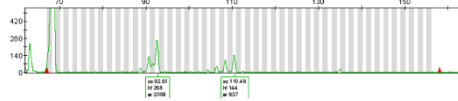

II-2

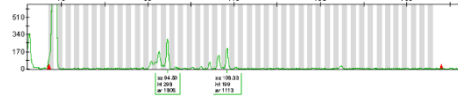

III-1

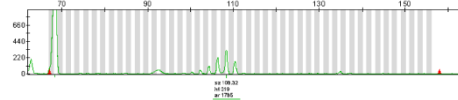

III-2

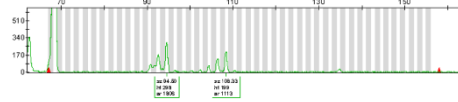

III-3

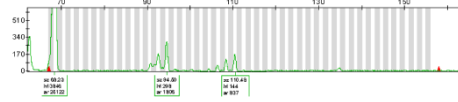

## TH

I-1

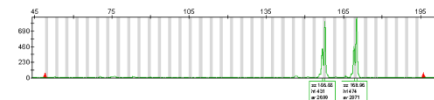

II-1

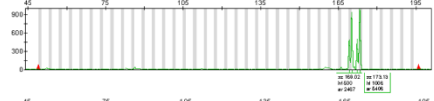

II-2

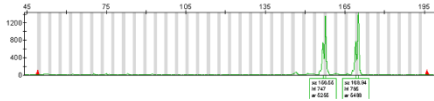

III-1

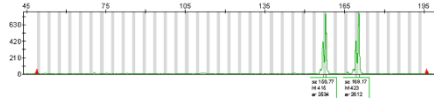

III-2

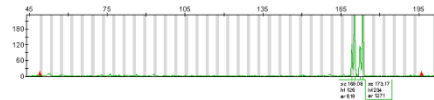

III-3

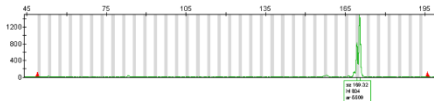

## D11S4046

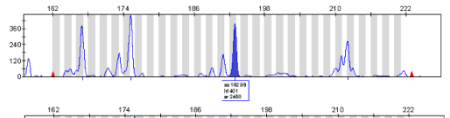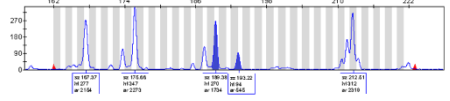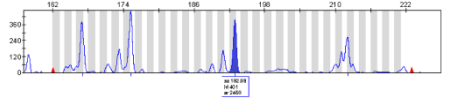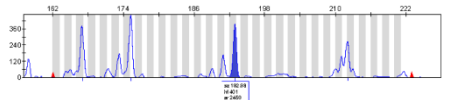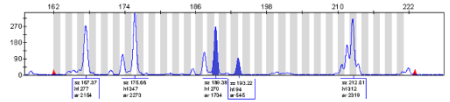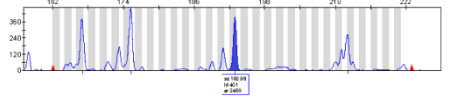

## D11S4088

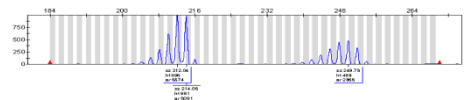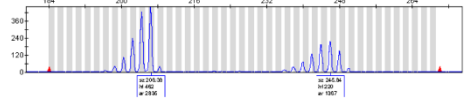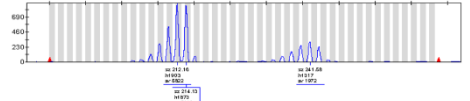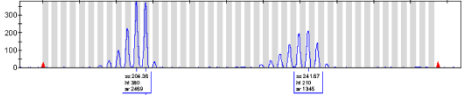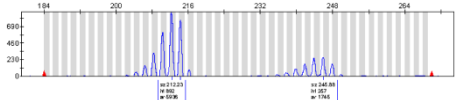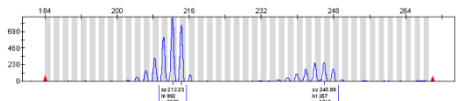

Supplement: Additional file 3: Figure S3. — Segregation of the haplotype associated with the duplication in family 1. Note that the haplotype associated with the duplication segregates from I-1 to II-2, III-2, and III-3 and that only the D11S4088 marker shows allelic imbalances in II-2, III-2, and III-3. (PDF 208 kb) [file 13148_2016_236_MOESM3_ESM.pdf]

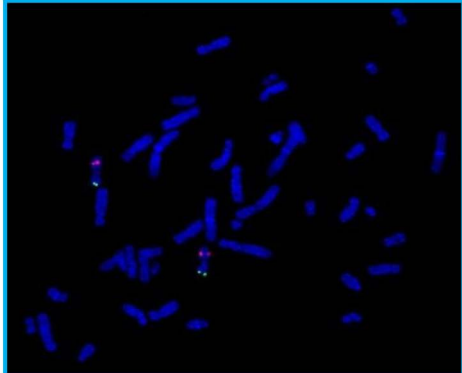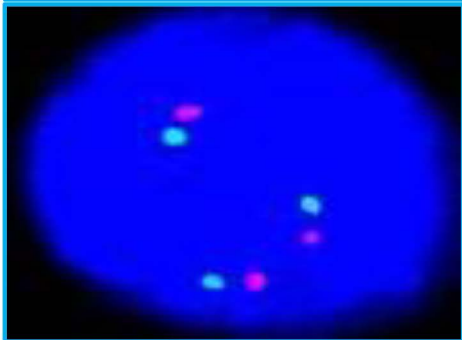

Additional file 4: Figure 4

Supplement: Additional file 4: Figure S4. — FISH analysis on metaphase nuclei (top panel) of cultured cells derived from the umbilical cord of the proband of family 1 by using BAC probes for 11p15.5-15.4 (RP11-81K4, 2798699-2970438, green) and 11q22.3 (RP11-876C12, 103,804,669-103,982,517, red). The green signal on both homologues is visible only at chr11p, demonstrating the presence of an in cis duplication and excluding an unbalanced translocation. FISH analysis on interphase nuclei (bottom panel) using the BACs RP11-11A9 (3,236,552-3,356,012, green) and RP11-81K4 (red), hybridizing within the duplication. Note that single and duplicated signals can be seen on the two homologues, respectively. The green-red-red-green order of the duplicated signals indicates that the duplication is inverted. (PDF 51 kb) [file 13148_2016_236_MOESM4_ESM.pdf]

# H19DMR/ICR1

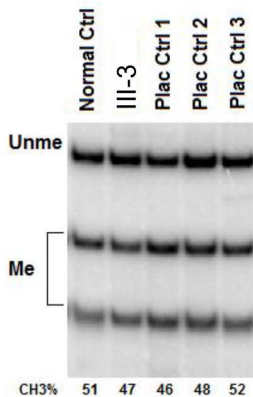

# KVDMR1/ICR2

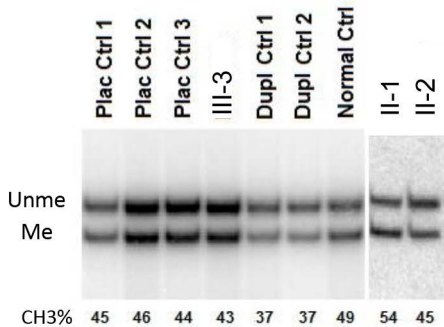

Additional file 5: Figure 5

Supplement: Additional file 5: Figure S5. — DNA methylation analysis of ICR1 and ICR2 in family 1, determined by combined bisulphite restriction assay (COBRA). The placenta DNAs of the proband and three healthy controls, the peripheral blood DNAs of the proband parents, one control, and two BWS patients (Dupl Ctrl) carrying a duplicated unmethylated ICR2 [15, 16]. Note that the proband (III-3) and his parents show normal methylation at both ICR1 and ICR2. Unme = non-methylated band, me = methylated band. (PDF 66 kb) [file 13148_2016_236_MOESM5_ESM.pdf]

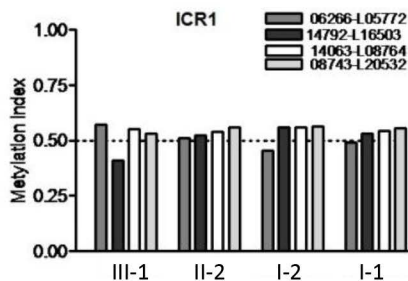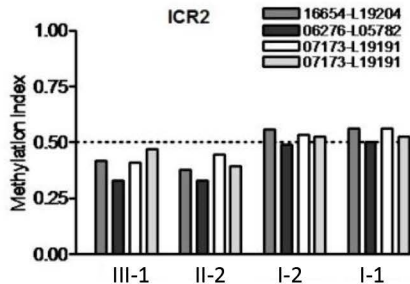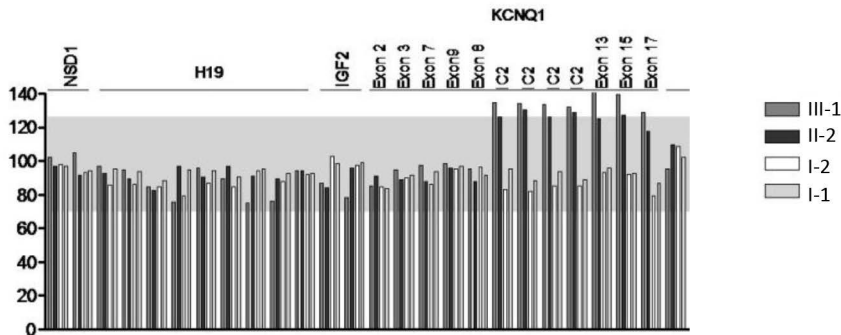

Additional file 6: Figure S6

Supplement: Additional file 6: Figure S6. — DNA methylation (top) and copy number (CN, bottom) analyses at 11p15 region in family 2, determined by MS-MLPA. The histograms represent the normalized DNA methylation of ICR1 and ICR2 and CN of the genomic region spanning from the NSD1 to KCNQ1 gene. The CN range that is considered normal is shadowed. Note that methylation of ICR2 is abnormally low while CN values of ICR2 and KCNQ1 exon 13-17 are abnormally high in the proband and his mother. (PDF 130 kb) [file 13148_2016_236_MOESM6_ESM.pdf]

D11S922

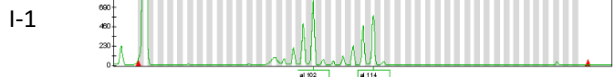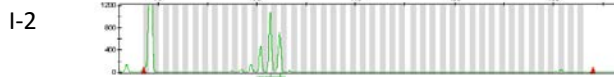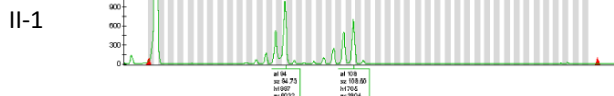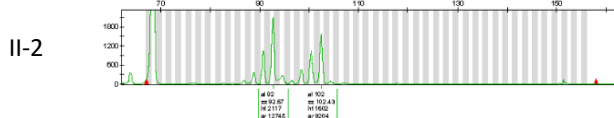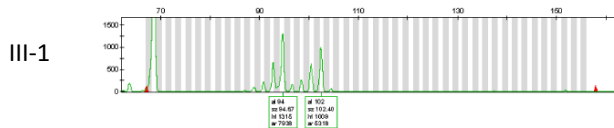

TH

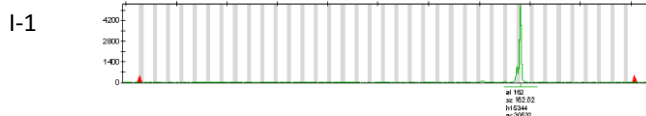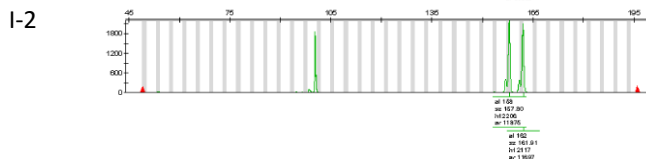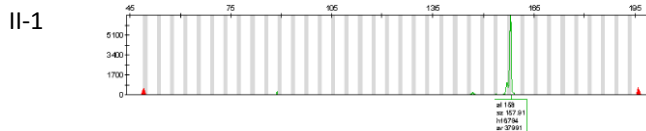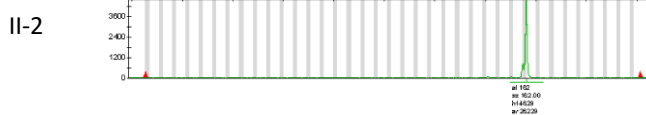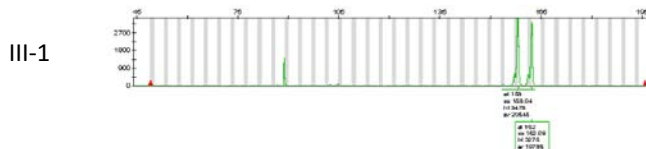

D11S4046

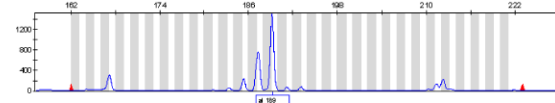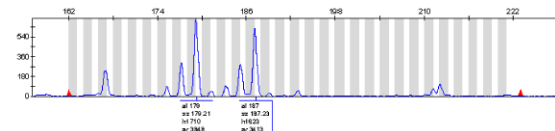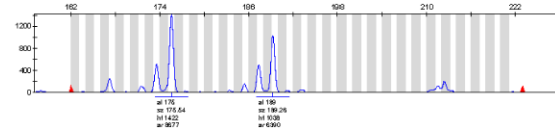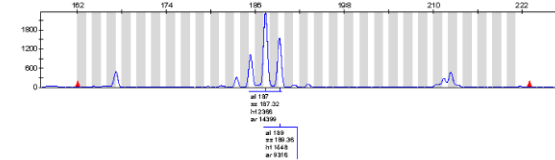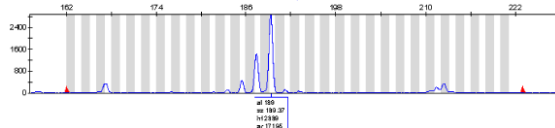

D11S4088

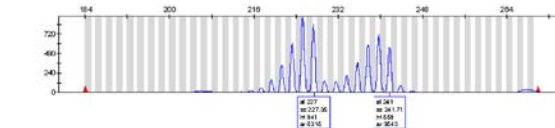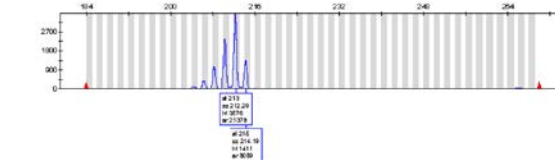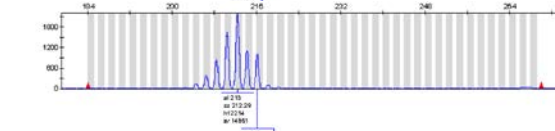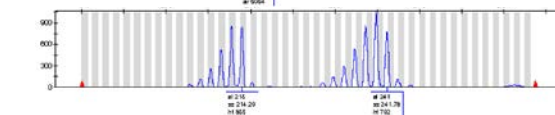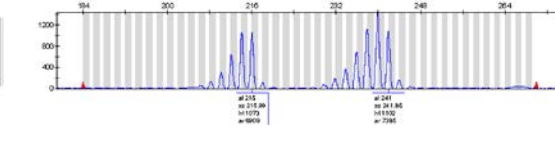

Supplement: Additional file 7: Figure S7. — Note that the haplotype associated with the duplication segregates from I-1 to II-2 and III-1 and that only the D11S4088 marker shows allelic imbalances in II-2 and III-1. (PDF 141 kb) [file 13148_2016_236_MOESM7_ESM.pdf]

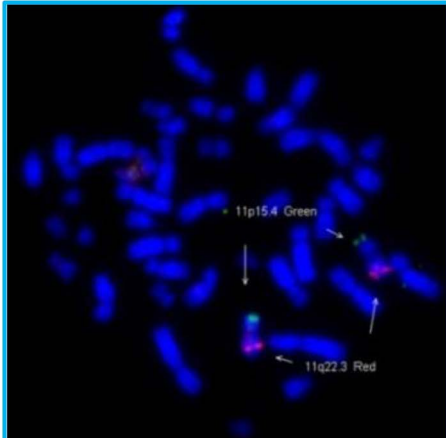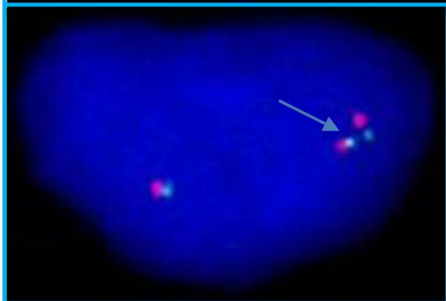

Additional file 8: Figure 8

Supplement: Additional file 8: Figure S8. — FISH analysis on metaphase nuclei (top panel) of cultured cells derived from peripheral blood leukocytes of the proband of family 2 by using BAC probes for 11p15.5-15.4 (RP11-11A9, 3,236,552-3,356,012, green) and 11q22.3 (RP11-179B7, 104,298,339-104,459,797, red). The green signal on both homologues is visible only at chr11p, demonstrating the presence of an in cis duplication and excluding an unbalanced translocation. FISH analysis on interphase nuclei (bottom panel) using the BACs RP11-699D10 (2.9–3.0 Mb, red) and RP11-11A9 (green), hybridizing within the duplication. Note that single and duplicated signals can be seen on the two homologues, respectively. The red-green-green-red order of the duplicated signals indicates that the duplication is inverted. (PDF 52 kb) [file 13148_2016_236_MOESM8_ESM.pdf]
